# Structural, Hirshfeld surface and three-dimensional inter­action-energy studies of 1,3,5-triethyl 2-amino-3,5-di­cyano-4,6-bis­(4-fluoro­phen­yl)cyclo­hex-1-ene-1,3,5-tri­carboxyl­ate

**DOI:** 10.1107/S2056989023003134

**Published:** 2023-04-14

**Authors:** S. N. Chandana, D. P. Ganesha, N. R. Sreenatha, A. S. Harisha, B. N. Lakshminarayana

**Affiliations:** a Department of Physics, Adichunchanagiri Institute of Technology, Chikkamagaluru 577102, Karnataka, India; b Department of Physics, Rajeev Institute of Technology, Hassan 573201, Karnataka, India; c Department of Physics, Government Engineering College, Bedarapura, Chamarajanagara 571313, Karnataka, India; d Alkem Laboratories Ltd, R&D Centre, Industrial Estate, 4th Phase, Bangalore, Karnataka, India; Universidade de Sâo Paulo, Brazil

**Keywords:** single-crystal XRD, envelope conformation, Hirshfeld surfaces, three-dimensional inter­action energies

## Abstract

In the title compound, C_29_H_27_F_2_N_3_O_6_, which crystallizes in the monoclinic space group *P*2_1_/*c*, the cyclo­hexenone ring is puckered and adopts an envelope conformation. The crystal structure features various inter­molecular inter­actions, such as N—H⋯O, C—H⋯N and C—H⋯O. These inter­actions were investigated using Hirshfeld surface analysis and the three-dimensional inter­action energies were calculated using the B3LYP/6–31 G(d,p) energy density model.

## Abstract 2

The various inter­molecular inter­actions, such as N—H⋯O, C—H⋯N and C—H⋯O, were investigated using Hirshfeld surface analysis and the three-dimensional inter­action energies were calculated.

## 1. Chemical context

Organic compounds containing hetero atoms such as fluorine, nitro­gen, sulfur and oxygen exhibit significant biological activities such as anti­oxidant (Fu *et al.*, 2010 ▸), insecticidal (Carbonnelle *et al.*, 2005 ▸), anti­bacterial, anti­fungal (Sener *et al.*, 2000 ▸), anti-inflammatory (Khanum *et al.*, 2004 ▸), anti­convulsant, analgesic and anti­tumor (Kushwaha *et al.*, 2011 ▸). These compounds find a wide range of applications in the fields of agriculture and biochemistry as well as in the pharmaceuticals industry. Hence, hetero organic compounds have attracted the attention of chemists with the aim of designing and synthesizing new organic compounds. The title compound was synthesized, its structure was studied by X-ray diffraction techniques and a computational analysis was performed to understand the inter­molecular inter­actions. 
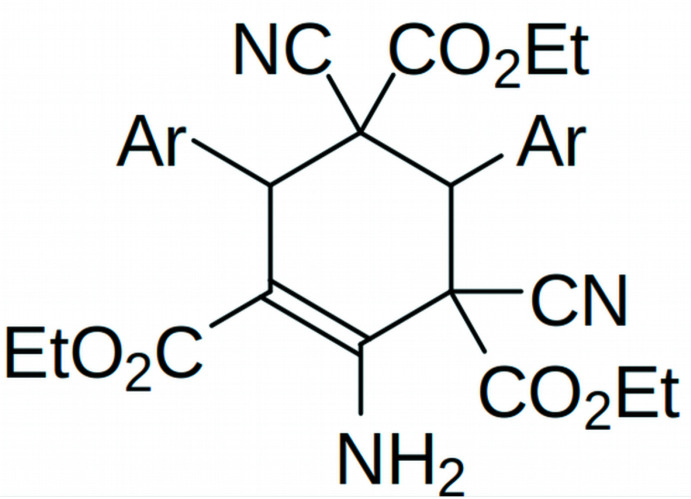


## 2. Structural commentary

**Figure 1:**
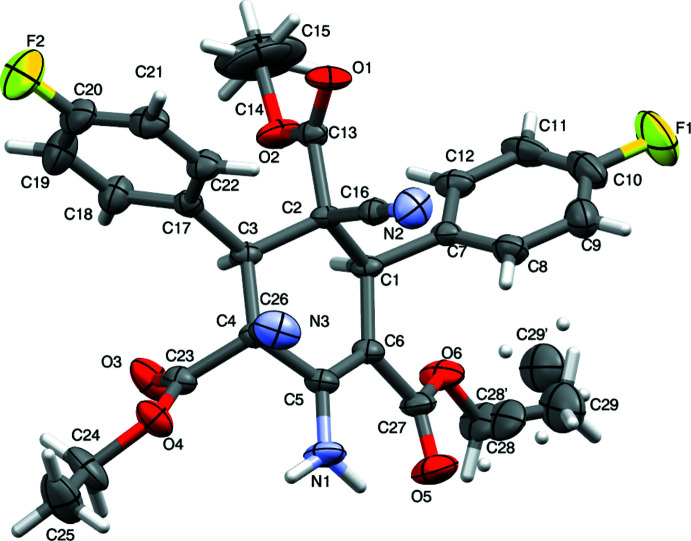
View of the title mol­ecule with displacement ellipsoids drawn at 40% probability level.

In the title compound (Fig. 1 ▸), the cyclo­hexenone ring (C1–C6) is puckered [maximum puckering amplitude *Q* = 0.554 (4) (3) Å and exhibits an envelope conformation on atom C2 (Cremer & Pople, 1975 ▸). The bond lengths and bond angles agree with those of previously reported related compounds (Gunasekaran *et al.*, 2009 ▸; Mertsalov *et al.*, 2021 ▸; Chandana *et al.*, 2021 ▸; Ganesha, Sreenatha *et al.*, 2023 ▸; Ganesha, Nizamuddin *et al.*, 2023 ▸; Ganesha *et al.*, 2022 ▸; Sreenatha *et al.*, 2018 ▸, 2020 ▸, 2022 ▸; Lakshminarayana *et al.*, 2009 ▸, 2010 ▸, 2022 ▸; Madan Kumar *et al.*, 2018 ▸; HariPrasada *et al.*, 2023 ▸). The dihedral angle between the mean plane of the cyclo­hexenone (C1–C6) and fluoro­benzene rings (C7–C12 and C17–C22) are 62.3 (2) and 84.9 (2)°, respectively, confirming the non-planarity of the mol­ecule and also the equatorial orientation of the rings. The carboxyl­ate group at the C2 position is oriented *+syn-clinical*, -*-anti-clinical*, *+anti-clinical* and *–syn-clinical* to the mean plane of the C1–C6 ring with torsion angles C1—C2—C13—O2 = 50.3 (4)°, C1—C2—C13—O1 = −131.3 (4)°, C3—C2—C13—O1 = 110.6 (4)° and C3—C2—C13—O2 = −67.8 (4)°. The orientation of other two carboxyl­ate groups at the C4 and C5 positions are described by the torsion angles C1—C6—C27—O6 = −15.4 (5)° (*–syn-periplanar*), C1—C6—C27—O5 = 167.2 (4)° (*+anti-periplanar*), C5—C6—C27—O5 = −17.2 (6)° (*–anti-periplanar*), C5—C6—C27—O6 = 160.1 (3)° (*+anti-periplanar*) and C3—C4—C23—O3 = 44.9 (5)° (*+syn-clinal*), C3—C4—C23—O4 = −136.4 (3)° (*–anti-clinal*), C5—C4—C23—O3 = −75.8 (4)° (*–syn-clinal*), C5—C4—C23—O4 = 102.9 (3)° (*+anti-clinal*). The orientation is due to the inter­molecular N—H⋯O and C—H⋯O inter­actions.

## 3. Supra­molecular features

**Figure 2:**
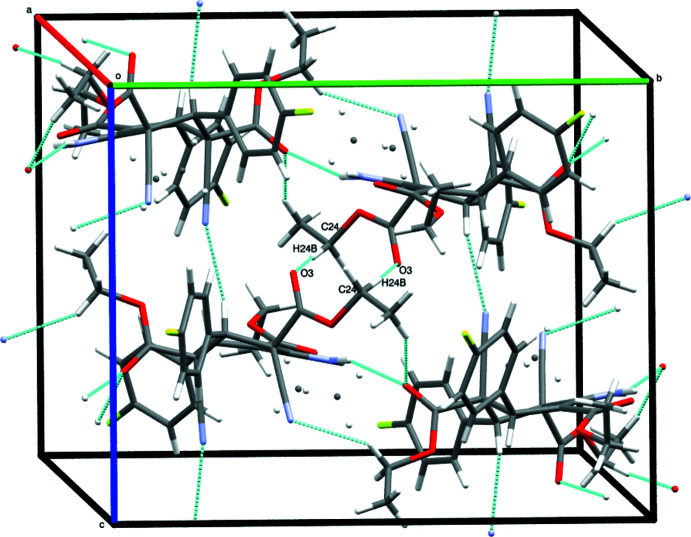
Packing of the mol­ecules along the *b* axis, showing the 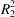 (10) ring motif.

In the crystal, the mol­ecules are held together by an inter­molecular inter­actions of the types N1—H2*N*⋯O1, C14—H14*B*⋯N3, and C24—H24*B*⋯O3 (Table 1 ▸), enclosing an 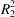 (10) closed ring motif, propagating along the [101] direction (Figs. 2 ▸ and 3 ▸).

## 4. Database survey

A survey of the Cambridge Structural Database (CSD version 5.41, update of November 2022; Groom *et al.*, 2016 ▸) reveals one nearly comparable derivative, triethyl 2-(5-nitro-2*H*-indazol-2-yl)propane-1,2,3-tri­carboxyl­ate (NUPQAS; Boulhaoua *et al.*, 2015 ▸) in which intermolecular C—H⋯O and C—H⋯N bonds are observed.

## 5. Hirshfeld surfaces and 2D fingerprint calculations

**Figure 4:**
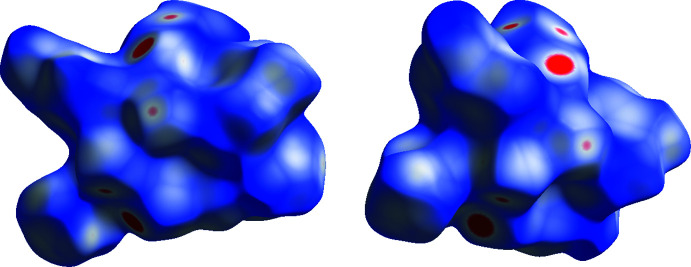
Hirshfeld surface mapped over *d*_norm_ (front and back views are shown).

**Figure 5:**
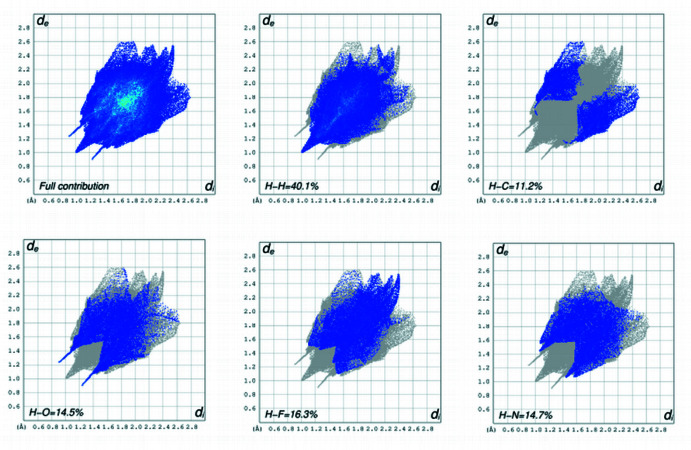
Two-dimensional fingerprint plots showing the pecentage contributions of various inter­atomic contacts.

The Hirshfeld surface (HS) mapped over *d*_norm_ was generated using *CrystalExplorer17.5* (Spackman *et al.*, 2009 ▸) with a colour scale of −0.3124 a.u. for red to +1.7877 a.u. for blue. The area and volume of the *d*_norm_ surface are 681.46 Å^2^ and 527.71 Å^3^, respectively. The front and rear views of the Hirshfeld surface mapped over *d*_norm_ are depicted in Fig. 4 ▸. The bright-red circular spots on *d*_norm_ indicates the presence of inter­molecular N1—H2*N*⋯O1, C14—H14*B*⋯N3 and C24—H24*B*⋯O3 inter­actions. The percentage contribution from different inter­molecular inter­actions towards the formation of a three dimensional Hirshfeld surface (HS) was computed using two-dimensional fingerprint calculations (Fig. 5 ▸). The results showed that the H⋯H (40.1%) contacts make the major contribution to the crystal packing, while the C⋯H (11.2%), N⋯H (14.7%), H⋯F (16.3%), H⋯O (14.5%) contacts also make a significant contribution to the total area of the HS surface.

## 6. Three-dimensional-framework analysis of inter­action energies

**Figure 6:**
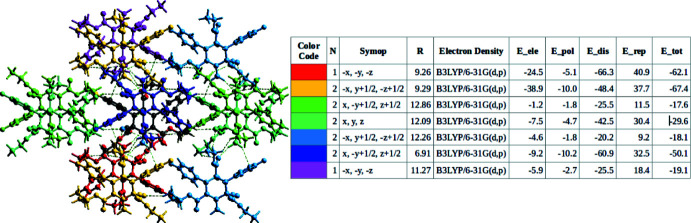
Visualization of the inter­action energy values between the reference mol­ecule and the constituents of a cluster within the default radius of 3.8 Å. The table gives information on the number of mol­ecules (*N*) inter­acting with the reference nolecule in a cluster, the rotational symmetry (*Symop*) and the corresponding mol­ecular centroid–centroid distances (*R*, in Å) and the inter­action energies in component form.

**Figure 7:**
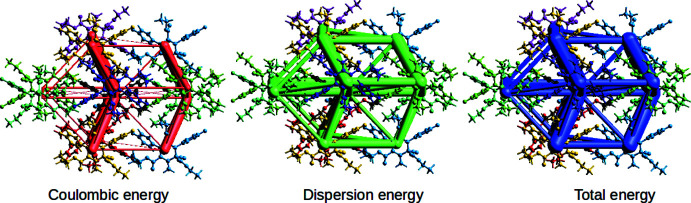
Three-dimensional energy frameworks of Coulombic, dispersion and total energy terms.

*CrystalExplorer 17.5* software calculates inter­action energies between crystal mol­ecular pairs. Energy calculations were carried out using the B3LYP/6-31G(d,p) basis set within a default radius of 3.8 Å (Turner *et al.*, 2015 ▸, 2017 ▸; Gavezzotti, 2002 ▸; Grimme, 2006 ▸). The interaction of different molecules with the reference mol­ecule (black ball-and-stick model at the centre) in the cluster of energy frameworks is depicted in Fig. 6 ▸. Fig. 7 ▸ depicts the energy frameworks, visualizing the strength of the inter­actions, with the Coulombic, dispersion and total energies shown in red, green and blue, respectively. The radii of the cylinders connecting the centroids of the mol­ecules indicate the relative strengths of the inter­action energies. A table of inter­action energies in component form is given in the table in Fig. 6 ▸. The highest total inter­action energy (*E*_tot_ = −67.4 kJ mol^−1^) is associated with a pair of yellow mol­ecules with the short centroid distance *R* = 9.29 Å with rotational symmetry −*x*, *y* + 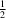 , −*z* + 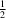 , while the lowest total inter­action energy (*E*_tot_ = −17.6 kJ mol^−1^) was observed for a pair of green mol­ecules inter­acting at the longer centroid distance *R* = 12.86 Å; this is in accordance with the classical laws of electrostatics. In each of the energy terms, the dispersion component is dominant over the others.

## 7. Synthesis and crystallization

Piperidine (6 mmol) was added to ethyl cyano­acetate (30 mmol) and the mixture was stirred for 10 min. Then 4-fluoro­benzaldehyde (20 mmol) was added dropwise and during the addition, the temperature of the reaction mass rose to 333 K (it should not be cooled), and the mass was stirred for 30 min. The temperature slowly came down to 293–298 K over 30 min. The progress of the reaction was monitored by TLC and found to be complete. Methyl­ene chloride (30 ml) and water (20 ml) were added and the mixture was stirred for 10 min. The organic layer was separated and washed with sat. aq. NaCl solution and dried over anhydrous Na_2_SO_4_, then concentrated under reduced pressure to get the crude product. This was purified by silica gel column chromatography using *n*-hepta­ne/ethyl acetate as eluent. The mixture was quenched in cold water and the organic layer was extracted with ethyl acetate, washed with 5% sodium bicarbonate solution, and dried over anhydrous sodium sulfate. Slow evaporation of the solvent lead to crystals of the title compound, which were recrystallized from ethanol solution.

## 8. Refinement

Crystal data, data collection and structure refinement details are summarized in Table 2 ▸. H atoms were placed at idealized positions and allowed to ride on their parent atoms with C—H distances in the range 0.93–0.98 Å and *U*_iso_(H) = 1.2*U*_eq_(C) (1.5 for methyl H atoms).

## Supplementary Material

Crystal structure: contains datablock(s) global, I. DOI: 10.1107/S2056989023003134/ex2065sup1.cif

Structure factors: contains datablock(s) I. DOI: 10.1107/S2056989023003134/ex2065Isup2.hkl

Supporting information file. DOI: 10.1107/S2056989023003134/ex2065Isup3.cml

CCDC reference: 2202315

Additional supporting information:  crystallographic information; 3D view; checkCIF report

## Figures and Tables

**Figure 3:**
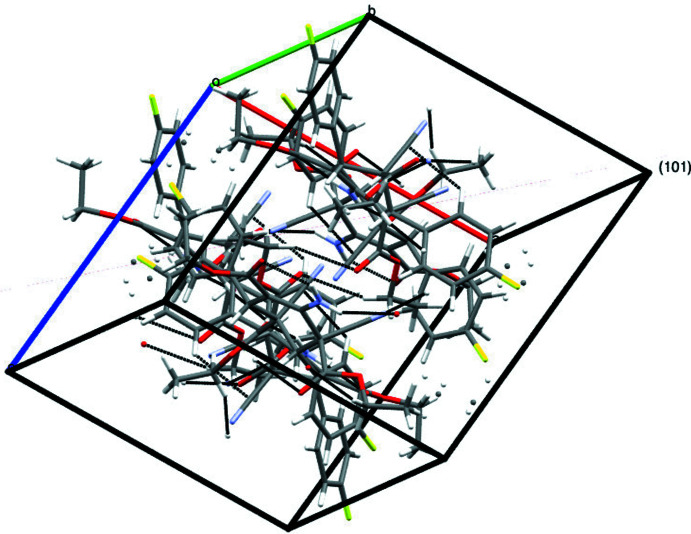
The inter­molecular inter­actions enclosing the 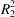 (10) ring motif propagating along the [101] direction.

**Table 1:** Hydrogen-bond geometry (Å, °)

*D*—H⋯*A*	*D*—H	H⋯*A*	*D*⋯*A*	*D*—H⋯*A*
C14—H14*B*⋯N3^i^	0.97	2.60	3.420 (7)	143
C24—H24*B*⋯O3^ii^	0.97	2.58	3.483 (6)	156
N1—H1*N*⋯O5	0.87 (2)	2.03 (4)	2.662 (5)	128 (4)
N1—H2*N*⋯O1^iii^	0.88 (2)	2.25 (3)	3.064 (4)	155 (4)
N1—H2*N*⋯O4	0.88 (2)	2.61 (4)	3.152 (5)	121 (4)

Symmetry codes: (i) 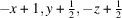 ; (ii) 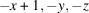 ; (iii) 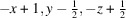 .

**Table 2:** Experimental details

Crystal data	
Chemical formula	C_29_H_27_F_2_N_3_O_6_
*M*_r_	551.53
Crystal system, space group	Monoclinic, *P*2_1_/*c*
Temperature (K)	297
*a*, *b*, *c* (Å)	12.0884 (11), 17.0492 (16), 13.5966 (11)
β (°)	100.008 (3)
*V* (Å^3^)	2759.6 (4)
*Z*	4
Radiation type	Mo *K*α
μ (mm^−1^)	0.10
Crystal size (mm)	0.14 × 0.09 × 0.04

Data collection	
Diffractometer	Bruker Kappa APEXIII PHOTON II
No. of measured, independent and observed [*I* > 2σ(*I*)] reflections	52965, 4869, 3477
*R*_int_	0.141
(sin θ/λ)_max_ (Å^−1^)	0.595

Refinement	
*R*[*F* ^2^ > 2σ(*F* ^2^)], *wR*(*F* ^2^), *S*	0.087, 0.214, 1.14
No. of reflections	4869
No. of parameters	386
No. of restraints	47
H-atom treatment	H atoms treated by a mixture of independent and constrained refinement
Δρ_max_, Δρ_min_ (e Å^−3^)	0.32, −0.31

Computer programs: *APEX3* and *SAINT* (Bruker, 2014 ▸), *SHELXT2018/2* (Sheldrick, 2015*a* ▸), *SHELXL2018/3* (Sheldrick, 2015*b* ▸) and *PLATON* (Spek, 2020 ▸).

## supplementary crystallographic information

### Crystal data

**Table:**

C_29_H_27_F_2_N_3_O_6_	*F*(000) = 1152
*M**_r_* = 551.53	*D*_x_ = 1.328 Mg m^−^^3^
Monoclinic, *P*2_1_/*c*	Mo *K*α radiation, λ = 0.71073 Å
*a* = 12.0884 (11) Å	Cell parameters from 3477 reflections
*b* = 17.0492 (16) Å	θ = 2.9–25.1°
*c* = 13.5966 (11) Å	µ = 0.10 mm^−^^1^
β = 100.008 (3)°	*T* = 297 K
*V* = 2759.6 (4) Å^3^	Block, colorless
*Z* = 4	0.14 × 0.09 × 0.04 mm

### Data collection

**Table:**

Bruker Kappa APEXIII PHOTON II diffractometer	*R*_int_ = 0.141
Radiation source: fine focus sealed tube	θ_max_ = 25.0°, θ_min_ = 2.9°
φ and ω scans	*h* = −14→14
52965 measured reflections	*k* = −20→20
4869 independent reflections	*l* = −16→15
3477 reflections with *I* > 2σ(*I*)	

### Refinement

**Table:**

Refinement on *F*^2^	47 restraints
Least-squares matrix: full	Hydrogen site location: mixed
*R*[*F*^2^ > 2σ(*F*^2^)] = 0.087	H atoms treated by a mixture of independent and constrained refinement
*wR*(*F*^2^) = 0.214	*w* = 1/[σ^2^(*F*_o_^2^) + (0.095*P*)^2^ + 2.1236*P*] where *P* = (*F*_o_^2^ + 2*F*_c_^2^)/3
*S* = 1.14	(Δ/σ)_max_ < 0.001
4869 reflections	Δρ_max_ = 0.32 e Å^−^^3^
386 parameters	Δρ_min_ = −0.30 e Å^−^^3^

### Special details

**Table:**

Geometry. All esds (except the esd in the dihedral angle between two l.s. planes) are estimated using the full covariance matrix. The cell esds are taken into account individually in the estimation of esds in distances, angles and torsion angles; correlations between esds in cell parameters are only used when they are defined by crystal symmetry. An approximate (isotropic) treatment of cell esds is used for estimating esds involving l.s. planes.

### Fractional atomic coordinates and isotropic or equivalent isotropic displacement parameters (Å^2^)

**Table:**

	*x*	*y*	*z*	*U*_iso_*/*U*_eq_	Occ. (<1)
C1	0.7198 (3)	0.2207 (2)	0.2001 (3)	0.0240 (8)	
H1	0.727769	0.231041	0.130783	0.029*	
C2	0.6104 (3)	0.2626 (2)	0.2181 (2)	0.0235 (8)	
C3	0.5101 (3)	0.2230 (2)	0.1488 (2)	0.0234 (8)	
H3	0.532115	0.221020	0.082740	0.028*	
C4	0.4967 (3)	0.1361 (2)	0.1785 (3)	0.0253 (8)	
C5	0.6106 (3)	0.0939 (2)	0.2085 (3)	0.0285 (8)	
C6	0.7102 (3)	0.1323 (2)	0.2112 (3)	0.0264 (8)	
C7	0.8219 (3)	0.2567 (2)	0.2671 (3)	0.0286 (8)	
C8	0.8565 (3)	0.2338 (2)	0.3650 (3)	0.0403 (10)	
H8	0.817146	0.194409	0.391282	0.048*	
C9	0.9480 (4)	0.2679 (3)	0.4252 (4)	0.0531 (12)	
H9	0.970380	0.252113	0.491150	0.064*	
C10	1.0048 (4)	0.3258 (3)	0.3841 (4)	0.0601 (14)	
C11	0.9741 (4)	0.3508 (3)	0.2875 (4)	0.0521 (13)	
H11	1.013665	0.390343	0.261732	0.062*	
C12	0.8822 (3)	0.3153 (2)	0.2293 (3)	0.0378 (10)	
H12	0.860441	0.331096	0.163341	0.045*	
C13	0.6100 (3)	0.3501 (2)	0.1913 (3)	0.0290 (8)	
C14	0.6296 (6)	0.4404 (3)	0.0641 (5)	0.085 (2)	
H14A	0.553966	0.451275	0.029724	0.102*	
H14B	0.646142	0.476250	0.120125	0.102*	
C15	0.7038 (9)	0.4537 (4)	−0.0001 (7)	0.143 (4)	
H15A	0.697339	0.507096	−0.022647	0.215*	
H15B	0.779025	0.444078	0.033956	0.215*	
H15C	0.686851	0.419104	−0.056445	0.215*	
C16	0.6009 (3)	0.2580 (2)	0.3251 (3)	0.0282 (8)	
C17	0.3999 (3)	0.2679 (2)	0.1335 (3)	0.0255 (8)	
C18	0.3455 (3)	0.2816 (3)	0.0369 (3)	0.0428 (11)	
H18	0.378204	0.264155	−0.016208	0.051*	
C19	0.2441 (4)	0.3203 (3)	0.0172 (4)	0.0556 (13)	
H19	0.208054	0.328936	−0.048125	0.067*	
C20	0.1976 (3)	0.3458 (3)	0.0969 (4)	0.0489 (12)	
C21	0.2487 (3)	0.3355 (2)	0.1932 (4)	0.0430 (11)	
H21	0.215853	0.354255	0.245598	0.052*	
C22	0.3500 (3)	0.2967 (2)	0.2117 (3)	0.0352 (9)	
H22	0.385888	0.289508	0.277302	0.042*	
C23	0.4342 (3)	0.0933 (2)	0.0843 (3)	0.0303 (9)	
C24	0.2967 (4)	−0.0024 (3)	0.0223 (4)	0.0538 (13)	
H24A	0.268639	0.030494	−0.034869	0.065*	
H24B	0.348193	−0.040468	0.002475	0.065*	
C25	0.2039 (5)	−0.0422 (4)	0.0563 (4)	0.0697 (16)	
H25A	0.164771	−0.074136	0.003303	0.105*	
H25B	0.232526	−0.074708	0.112636	0.105*	
H25C	0.153279	−0.004020	0.075435	0.105*	
C26	0.4322 (3)	0.1278 (2)	0.2620 (3)	0.0312 (9)	
C27	0.8120 (3)	0.0845 (2)	0.2178 (3)	0.0356 (9)	
C28	0.998 (3)	0.084 (2)	0.1734 (17)	0.058 (5)	0.345 (12)
H28A	1.029815	0.108563	0.120192	0.070*	0.345 (12)
H28B	0.984697	0.029090	0.158581	0.070*	0.345 (12)
C29	1.0710 (15)	0.0958 (13)	0.2720 (15)	0.084 (5)	0.345 (12)
H29A	1.142674	0.071719	0.271532	0.126*	0.345 (12)
H29B	1.081077	0.150867	0.285153	0.126*	0.345 (12)
H29C	1.036386	0.072146	0.323178	0.126*	0.345 (12)
C28'	1.0074 (13)	0.0836 (10)	0.2141 (11)	0.063 (3)	0.655 (12)
H28C	1.016442	0.061117	0.280600	0.076*	0.655 (12)
H28D	1.013141	0.041797	0.166848	0.076*	0.655 (12)
C29'	1.0950 (6)	0.1438 (6)	0.2095 (8)	0.070 (3)	0.655 (12)
H29D	1.167949	0.120150	0.225539	0.105*	0.655 (12)
H29E	1.085169	0.165536	0.143452	0.105*	0.655 (12)
H29F	1.088455	0.184765	0.256673	0.105*	0.655 (12)
F1	1.0955 (3)	0.3596 (2)	0.4425 (3)	0.0998 (13)	
F2	0.0967 (2)	0.3833 (2)	0.0775 (3)	0.0818 (10)	
N1	0.5985 (3)	0.01657 (19)	0.2255 (3)	0.0421 (9)	
H1N	0.658 (3)	−0.013 (2)	0.240 (3)	0.050*	
H2N	0.536 (2)	−0.010 (2)	0.215 (3)	0.050*	
N2	0.5956 (3)	0.2536 (2)	0.4077 (3)	0.0475 (10)	
N3	0.3848 (3)	0.1197 (2)	0.3262 (3)	0.0478 (10)	
O1	0.5874 (3)	0.40133 (16)	0.2450 (2)	0.0477 (8)	
O2	0.6346 (3)	0.36003 (16)	0.1017 (2)	0.0444 (8)	
O3	0.4589 (3)	0.10243 (19)	0.0037 (2)	0.0515 (9)	
O4	0.3547 (2)	0.04598 (17)	0.10520 (19)	0.0394 (7)	
O5	0.8213 (3)	0.01601 (17)	0.2428 (3)	0.0567 (9)	
O6	0.8963 (2)	0.12367 (16)	0.1887 (2)	0.0464 (8)	

### Atomic displacement parameters (Å^2^)

**Table:**

	*U*^11^	*U*^22^	*U*^33^	*U*^12^	*U*^13^	*U*^23^
C1	0.0252 (19)	0.0198 (18)	0.0296 (19)	0.0023 (15)	0.0116 (15)	−0.0003 (15)
C2	0.0297 (19)	0.0174 (17)	0.0250 (18)	0.0007 (15)	0.0095 (15)	0.0000 (14)
C3	0.0286 (19)	0.0199 (18)	0.0238 (18)	0.0012 (15)	0.0109 (15)	−0.0016 (14)
C4	0.0258 (19)	0.0227 (19)	0.0297 (19)	0.0009 (15)	0.0112 (15)	0.0006 (15)
C5	0.035 (2)	0.0190 (18)	0.033 (2)	−0.0016 (16)	0.0098 (16)	−0.0033 (15)
C6	0.031 (2)	0.0200 (18)	0.0295 (19)	0.0007 (16)	0.0084 (15)	−0.0031 (15)
C7	0.0266 (19)	0.0179 (18)	0.042 (2)	0.0016 (15)	0.0066 (16)	−0.0019 (16)
C8	0.038 (2)	0.028 (2)	0.054 (3)	−0.0041 (18)	0.006 (2)	−0.0009 (19)
C9	0.043 (3)	0.055 (3)	0.057 (3)	0.001 (2)	−0.005 (2)	−0.010 (2)
C10	0.035 (3)	0.054 (3)	0.089 (4)	−0.007 (2)	0.003 (3)	−0.030 (3)
C11	0.035 (2)	0.031 (2)	0.094 (4)	−0.012 (2)	0.023 (3)	−0.015 (2)
C12	0.031 (2)	0.028 (2)	0.057 (3)	0.0002 (18)	0.0158 (19)	0.0007 (19)
C13	0.0242 (19)	0.0237 (19)	0.040 (2)	−0.0014 (16)	0.0065 (16)	−0.0012 (17)
C14	0.125 (5)	0.039 (3)	0.102 (5)	0.024 (3)	0.049 (4)	0.042 (3)
C15	0.218 (10)	0.061 (4)	0.180 (8)	0.010 (5)	0.113 (8)	0.055 (5)
C16	0.027 (2)	0.025 (2)	0.033 (2)	0.0031 (15)	0.0077 (16)	−0.0020 (16)
C17	0.0251 (19)	0.0210 (18)	0.031 (2)	−0.0038 (15)	0.0060 (15)	0.0016 (15)
C18	0.040 (2)	0.050 (3)	0.038 (2)	0.009 (2)	0.0066 (19)	0.002 (2)
C19	0.046 (3)	0.070 (3)	0.047 (3)	0.014 (3)	−0.002 (2)	0.011 (2)
C20	0.025 (2)	0.042 (3)	0.078 (4)	0.0076 (19)	0.001 (2)	0.004 (2)
C21	0.030 (2)	0.036 (2)	0.065 (3)	0.0027 (19)	0.016 (2)	−0.007 (2)
C22	0.031 (2)	0.034 (2)	0.042 (2)	0.0045 (18)	0.0097 (18)	−0.0013 (18)
C23	0.029 (2)	0.025 (2)	0.038 (2)	−0.0019 (16)	0.0098 (17)	−0.0040 (16)
C24	0.048 (3)	0.061 (3)	0.054 (3)	−0.024 (2)	0.013 (2)	−0.032 (2)
C25	0.064 (3)	0.075 (4)	0.068 (3)	−0.037 (3)	0.005 (3)	−0.005 (3)
C26	0.034 (2)	0.0210 (19)	0.040 (2)	−0.0043 (16)	0.0117 (18)	−0.0006 (16)
C27	0.036 (2)	0.019 (2)	0.052 (3)	0.0042 (17)	0.0066 (19)	−0.0006 (18)
C28	0.021 (6)	0.054 (6)	0.100 (12)	0.014 (5)	0.010 (9)	−0.010 (10)
C29	0.043 (8)	0.086 (10)	0.118 (11)	−0.001 (8)	0.000 (8)	−0.017 (9)
C28'	0.027 (5)	0.057 (4)	0.105 (10)	0.012 (4)	0.008 (7)	−0.008 (8)
C29'	0.031 (4)	0.075 (6)	0.105 (7)	−0.002 (4)	0.011 (4)	−0.003 (5)
F1	0.058 (2)	0.097 (3)	0.131 (3)	−0.0340 (19)	−0.0192 (19)	−0.035 (2)
F2	0.0386 (16)	0.087 (2)	0.115 (3)	0.0305 (16)	0.0012 (16)	−0.003 (2)
N1	0.036 (2)	0.0187 (18)	0.072 (3)	−0.0024 (15)	0.0125 (19)	0.0036 (17)
N2	0.056 (2)	0.055 (2)	0.034 (2)	0.0074 (19)	0.0148 (17)	0.0013 (17)
N3	0.060 (2)	0.046 (2)	0.045 (2)	−0.0092 (19)	0.030 (2)	−0.0024 (17)
O1	0.064 (2)	0.0228 (15)	0.059 (2)	0.0075 (14)	0.0186 (16)	−0.0112 (14)
O2	0.064 (2)	0.0279 (15)	0.0453 (17)	0.0084 (14)	0.0213 (15)	0.0111 (13)
O3	0.063 (2)	0.059 (2)	0.0383 (17)	−0.0270 (17)	0.0225 (15)	−0.0144 (15)
O4	0.0377 (16)	0.0429 (16)	0.0396 (16)	−0.0169 (13)	0.0127 (12)	−0.0084 (13)
O5	0.0423 (18)	0.0280 (17)	0.100 (3)	0.0084 (14)	0.0130 (17)	0.0077 (16)
O6	0.0277 (15)	0.0306 (15)	0.085 (2)	0.0050 (12)	0.0201 (15)	−0.0051 (15)

### Geometric parameters (Å, º)

**Table:**

C1—C6	1.520 (5)	C17—C22	1.399 (5)
C1—C7	1.530 (5)	C18—C19	1.377 (6)
C1—C2	1.559 (5)	C18—H18	0.9300
C1—H1	0.9800	C19—C20	1.375 (7)
C2—C16	1.480 (5)	C19—H19	0.9300
C2—C13	1.535 (5)	C20—C21	1.359 (6)
C2—C3	1.555 (5)	C20—F2	1.362 (5)
C3—C17	1.520 (5)	C21—C22	1.375 (6)
C3—C4	1.551 (5)	C21—H21	0.9300
C3—H3	0.9800	C22—H22	0.9300
C4—C26	1.491 (5)	C23—O3	1.194 (4)
C4—C5	1.545 (5)	C23—O4	1.322 (4)
C4—C23	1.552 (5)	C24—C25	1.453 (6)
C5—N1	1.351 (5)	C24—O4	1.472 (5)
C5—C6	1.365 (5)	C24—H24A	0.9700
C6—C27	1.466 (5)	C24—H24B	0.9700
C7—C8	1.380 (6)	C25—H25A	0.9600
C7—C12	1.387 (5)	C25—H25B	0.9600
C8—C9	1.385 (6)	C25—H25C	0.9600
C8—H8	0.9300	C26—N3	1.133 (5)
C9—C10	1.376 (7)	C27—O5	1.216 (5)
C9—H9	0.9300	C27—O6	1.334 (5)
C10—F1	1.365 (5)	C28—O6	1.45 (3)
C10—C11	1.369 (7)	C28—C29	1.484 (18)
C11—C12	1.386 (6)	C28—H28A	0.9700
C11—H11	0.9300	C28—H28B	0.9700
C12—H12	0.9300	C29—H29A	0.9600
C13—O1	1.200 (4)	C29—H29B	0.9600
C13—O2	1.314 (4)	C29—H29C	0.9600
C14—C15	1.375 (8)	C28'—C29'	1.484 (14)
C14—O2	1.460 (5)	C28'—O6	1.494 (18)
C14—H14A	0.9700	C28'—H28C	0.9700
C14—H14B	0.9700	C28'—H28D	0.9700
C15—H15A	0.9600	C29'—H29D	0.9600
C15—H15B	0.9600	C29'—H29E	0.9600
C15—H15C	0.9600	C29'—H29F	0.9600
C16—N2	1.139 (5)	N1—H1N	0.874 (19)
C17—C18	1.383 (5)	N1—H2N	0.875 (19)
			
C6—C1—C7	113.9 (3)	C22—C17—C3	123.8 (3)
C6—C1—C2	110.9 (3)	C19—C18—C17	121.7 (4)
C7—C1—C2	109.9 (3)	C19—C18—H18	119.1
C6—C1—H1	107.2	C17—C18—H18	119.1
C7—C1—H1	107.2	C20—C19—C18	118.0 (4)
C2—C1—H1	107.2	C20—C19—H19	121.0
C16—C2—C13	106.7 (3)	C18—C19—H19	121.0
C16—C2—C3	112.8 (3)	C21—C20—F2	119.2 (4)
C13—C2—C3	107.9 (3)	C21—C20—C19	122.6 (4)
C16—C2—C1	110.1 (3)	F2—C20—C19	118.1 (4)
C13—C2—C1	112.1 (3)	C20—C21—C22	118.6 (4)
C3—C2—C1	107.4 (3)	C20—C21—H21	120.7
C17—C3—C4	112.8 (3)	C22—C21—H21	120.7
C17—C3—C2	115.9 (3)	C21—C22—C17	121.2 (4)
C4—C3—C2	111.2 (3)	C21—C22—H22	119.4
C17—C3—H3	105.3	C17—C22—H22	119.4
C4—C3—H3	105.3	O3—C23—O4	125.7 (4)
C2—C3—H3	105.3	O3—C23—C4	122.1 (3)
C26—C4—C5	108.3 (3)	O4—C23—C4	112.1 (3)
C26—C4—C3	112.4 (3)	C25—C24—O4	108.0 (4)
C5—C4—C3	112.6 (3)	C25—C24—H24A	110.1
C26—C4—C23	109.9 (3)	O4—C24—H24A	110.1
C5—C4—C23	106.4 (3)	C25—C24—H24B	110.1
C3—C4—C23	107.0 (3)	O4—C24—H24B	110.1
N1—C5—C6	125.8 (4)	H24A—C24—H24B	108.4
N1—C5—C4	112.4 (3)	C24—C25—H25A	109.5
C6—C5—C4	121.7 (3)	C24—C25—H25B	109.5
C5—C6—C27	117.6 (3)	H25A—C25—H25B	109.5
C5—C6—C1	123.7 (3)	C24—C25—H25C	109.5
C27—C6—C1	118.6 (3)	H25A—C25—H25C	109.5
C8—C7—C12	118.0 (4)	H25B—C25—H25C	109.5
C8—C7—C1	122.6 (3)	N3—C26—C4	178.2 (4)
C12—C7—C1	119.3 (4)	O5—C27—O6	121.7 (4)
C7—C8—C9	121.9 (4)	O5—C27—C6	125.9 (4)
C7—C8—H8	119.0	O6—C27—C6	112.4 (3)
C9—C8—H8	119.0	O6—C28—C29	101.3 (19)
C10—C9—C8	117.7 (5)	O6—C28—H28A	111.5
C10—C9—H9	121.2	C29—C28—H28A	111.5
C8—C9—H9	121.2	O6—C28—H28B	111.5
F1—C10—C11	118.9 (5)	C29—C28—H28B	111.5
F1—C10—C9	118.3 (5)	H28A—C28—H28B	109.3
C11—C10—C9	122.8 (4)	C28—C29—H29A	109.5
C10—C11—C12	117.9 (4)	C28—C29—H29B	109.5
C10—C11—H11	121.0	H29A—C29—H29B	109.5
C12—C11—H11	121.0	C28—C29—H29C	109.5
C11—C12—C7	121.6 (4)	H29A—C29—H29C	109.5
C11—C12—H12	119.2	H29B—C29—H29C	109.5
C7—C12—H12	119.2	C29'—C28'—O6	107.0 (12)
O1—C13—O2	125.6 (4)	C29'—C28'—H28C	110.3
O1—C13—C2	123.7 (3)	O6—C28'—H28C	110.3
O2—C13—C2	110.7 (3)	C29'—C28'—H28D	110.3
C15—C14—O2	112.7 (5)	O6—C28'—H28D	110.3
C15—C14—H14A	109.1	H28C—C28'—H28D	108.6
O2—C14—H14A	109.1	C28'—C29'—H29D	109.5
C15—C14—H14B	109.1	C28'—C29'—H29E	109.5
O2—C14—H14B	109.1	H29D—C29'—H29E	109.5
H14A—C14—H14B	107.8	C28'—C29'—H29F	109.5
C14—C15—H15A	109.5	H29D—C29'—H29F	109.5
C14—C15—H15B	109.5	H29E—C29'—H29F	109.5
H15A—C15—H15B	109.5	C5—N1—H1N	120 (3)
C14—C15—H15C	109.5	C5—N1—H2N	127 (3)
H15A—C15—H15C	109.5	H1N—N1—H2N	113 (4)
H15B—C15—H15C	109.5	C13—O2—C14	116.3 (4)
N2—C16—C2	178.5 (4)	C23—O4—C24	116.4 (3)
C18—C17—C22	117.8 (3)	C27—O6—C28	121.5 (14)
C18—C17—C3	118.4 (3)	C27—O6—C28'	113.9 (6)
			
C6—C1—C2—C16	70.5 (4)	C8—C7—C12—C11	−0.5 (6)
C7—C1—C2—C16	−56.4 (4)	C1—C7—C12—C11	178.8 (3)
C6—C1—C2—C13	−171.0 (3)	C16—C2—C13—O1	−10.8 (5)
C7—C1—C2—C13	62.1 (4)	C3—C2—C13—O1	110.6 (4)
C6—C1—C2—C3	−52.6 (4)	C1—C2—C13—O1	−131.3 (4)
C7—C1—C2—C3	−179.6 (3)	C16—C2—C13—O2	170.8 (3)
C16—C2—C3—C17	74.3 (4)	C3—C2—C13—O2	−67.8 (4)
C13—C2—C3—C17	−43.3 (4)	C1—C2—C13—O2	50.3 (4)
C1—C2—C3—C17	−164.3 (3)	C4—C3—C17—C18	−102.1 (4)
C16—C2—C3—C4	−56.4 (4)	C2—C3—C17—C18	128.1 (4)
C13—C2—C3—C4	−173.9 (3)	C4—C3—C17—C22	78.1 (4)
C1—C2—C3—C4	65.1 (3)	C2—C3—C17—C22	−51.8 (5)
C17—C3—C4—C26	−49.1 (4)	C22—C17—C18—C19	−1.7 (6)
C2—C3—C4—C26	83.1 (4)	C3—C17—C18—C19	178.5 (4)
C17—C3—C4—C5	−171.8 (3)	C17—C18—C19—C20	0.3 (7)
C2—C3—C4—C5	−39.6 (4)	C18—C19—C20—C21	1.2 (7)
C17—C3—C4—C23	71.6 (3)	C18—C19—C20—F2	−179.2 (4)
C2—C3—C4—C23	−156.2 (3)	F2—C20—C21—C22	179.2 (4)
C26—C4—C5—N1	60.7 (4)	C19—C20—C21—C22	−1.2 (7)
C3—C4—C5—N1	−174.3 (3)	C20—C21—C22—C17	−0.3 (6)
C23—C4—C5—N1	−57.4 (4)	C18—C17—C22—C21	1.7 (6)
C26—C4—C5—C6	−122.6 (4)	C3—C17—C22—C21	−178.5 (4)
C3—C4—C5—C6	2.4 (5)	C26—C4—C23—O3	167.2 (4)
C23—C4—C5—C6	119.3 (4)	C5—C4—C23—O3	−75.7 (5)
N1—C5—C6—C27	10.0 (6)	C3—C4—C23—O3	44.9 (5)
C4—C5—C6—C27	−166.2 (3)	C26—C4—C23—O4	−14.0 (4)
N1—C5—C6—C1	−174.7 (4)	C5—C4—C23—O4	103.0 (3)
C4—C5—C6—C1	9.1 (5)	C3—C4—C23—O4	−136.4 (3)
C7—C1—C6—C5	142.1 (3)	C5—C6—C27—O5	−17.2 (6)
C2—C1—C6—C5	17.4 (5)	C1—C6—C27—O5	167.2 (4)
C7—C1—C6—C27	−42.7 (4)	C5—C6—C27—O6	160.1 (3)
C2—C1—C6—C27	−167.4 (3)	C1—C6—C27—O6	−15.4 (5)
C6—C1—C7—C8	−41.2 (5)	O1—C13—O2—C14	−2.4 (6)
C2—C1—C7—C8	84.0 (4)	C2—C13—O2—C14	176.0 (4)
C6—C1—C7—C12	139.5 (3)	C15—C14—O2—C13	150.1 (7)
C2—C1—C7—C12	−95.3 (4)	O3—C23—O4—C24	3.5 (6)
C12—C7—C8—C9	0.4 (6)	C4—C23—O4—C24	−175.1 (3)
C1—C7—C8—C9	−179.0 (4)	C25—C24—O4—C23	−172.8 (4)
C7—C8—C9—C10	−0.3 (7)	O5—C27—O6—C28	7.9 (11)
C8—C9—C10—F1	−179.7 (4)	C6—C27—O6—C28	−169.6 (10)
C8—C9—C10—C11	0.3 (7)	O5—C27—O6—C28'	−14.7 (9)
F1—C10—C11—C12	179.5 (4)	C6—C27—O6—C28'	167.8 (7)
C9—C10—C11—C12	−0.5 (7)	C29—C28—O6—C27	−94 (2)
C10—C11—C12—C7	0.6 (6)	C29'—C28'—O6—C27	−161.1 (8)

### Hydrogen-bond geometry (Å, º)

**Table:**

*D*—H···*A*	*D*—H	H···*A*	*D*···*A*	*D*—H···*A*
C14—H14*B*···N3^i^	0.97	2.60	3.420 (7)	143
C24—H24*B*···O3^ii^	0.97	2.58	3.483 (6)	156
N1—H1*N*···O5	0.87 (2)	2.03 (4)	2.662 (5)	128 (4)
N1—H2*N*···O1^iii^	0.88 (2)	2.25 (3)	3.064 (4)	155 (4)
N1—H2*N*···O4	0.88 (2)	2.61 (4)	3.152 (5)	121 (4)

Symmetry codes: (i) −*x*+1, *y*+1/2, −*z*+1/2; (ii) −*x*+1, −*y*, −*z*; (iii) −*x*+1, *y*−1/2, −*z*+1/2.
